# Increasing Screening Follow-Up for Vulnerable Children: A Partnership with School Nurses

**DOI:** 10.3390/ijerph15081572

**Published:** 2018-07-25

**Authors:** Eunice Rodriguez, Ashini Srivastava, Melinda Landau

**Affiliations:** 1 Department of Pediatrics; Stanford University, Stanford, CA 94305, USA; er23@stanford.edu (E.R.); ashini@stanford.edu (A.S.); 2 Health and Family Support Department, San Jose Unified School District, San Jose, CA 95126, USA

**Keywords:** school health, vision screening, school nurses, screening follow-up, school-based health clinic

## Abstract

Approximately 20% of school-age children have a vision problem. Screening is an effective way to detect visual impairments, although only if adequate follow-up is available. Here, we evaluate the impact of hiring full-time nurses in four underserved schools on the likelihood of increasing follow-up for treatment after vision screening. First, we compared descriptive screening follow-up data from the intervention schools with that of five matched schools with part-time nurses in San Jose, California, from 2008 to 2012. The intervention schools had around 2800 low-income, minority children each year, and the five comparison schools had around 3445. Secondly, we conducted a qualitative analysis of open-ended survey responses from 129 teachers in the nine participating schools. In the final year, 96% of the students screened and referred for possible vision problems in schools with full-time nurses were followed up and examined by a health care provider. Yet, only 67% of students screened in comparison schools were examined. Teachers in schools with full-time nurses reported that follow-up of vision problems and getting glasses for students was the most beneficial activity performed by the nurses. School nurses can effectively increase medical care coordination and follow-up of vision screening in low-income communities.

## 1. Introduction

Student enrollment in public schools across the United States is expected to reach 51.7 million by fall of 2026 [1], and according to recent estimates, 51% of students currently enrolled in public schools are from low-income families [2]. Evidence shows that healthier students are better learners [3]. Students from low socio-economic backgrounds have higher levels of poor health and unmet health needs which could contribute to lower educational achievement and widen an academic achievement gap in relation to higher socioeconomic groups with better access to health care and prevention services [4]. In the case of vision problems, 1 in 5 school-age children have a condition identifiable by screening, and 80% of vision problems can be corrected with glasses [5]. Nearly 50% of children from low-income families may have vision problems that interfere with their academic performance [6]. According to the Centers for Disease Control and Prevention (CDC) children from families with incomes below poverty level are less likely to see an eye care provider compared to children from families with incomes >200% of the federal poverty level (17% versus 23%) [7], and children lacking health insurance have greater unmet vision needs compared to children with vision insurance (23% versus 5%) [8].

The American Academy of Pediatrics (AAP) recommends screening tests for all school-age children as a way to promote health, detect disease, and prevent injury and future health problems [9]. To that effect, one of the objectives of Healthy People 2020 is to reduce visual impairment due to uncorrected refractive error by 10% from 136.1 to 122.5 per 1000 individuals in the population aged 12 years and older [10].

In this paper we first provide a brief review of school-based screening programs, and then we investigate the impact that having full-time nurses in schools can have in increasing follow-up to vision screening among low income and minority children. We use a nurse demonstration intervention in the San Jose Unified School District (SJUSD) in California as a case study, and also assess teachers’ perceptions on the program impact.

### 1.1. Background: School Health Screening in California

In the United States 40 states have mandated vision screening for children, but screening methods vary across the states [11]. In the state of California, certain health services are required for all students by California Education Code and other services are provided to certain students under the Individuals with Disability Act [12]. The California Department of Education supports schools by providing health and school nursing services for vision, hearing, and scoliosis screening [13]. Since 1947, it has been mandatory for governing boards of all school districts in California to provide vision screening for all students enrolled in public schools [14]. According to California’s Education Code, students are screened for vision at kindergarten, upon first enrollment or entry in California school district at elementary school level, and subsequently, in grades 2, 5, and 8 by the school nurse or other authorized personnel [15].

Refractive errors are the most common vision disorder in children and prevalence rates vary depending on type of diagnostic criteria and examination methods used. Based on data from the National Survey of Children’s Health 2011–2012, prevalence of myopia or nearsightedness among children aged 5–17 years was reported as 9%, hyperopia or farsightedness as 13%, and prevalence of astigmatism varied between 15% and 28%, depending on threshold of diagnosis used. Rates also varied across age, race, and ethnicity [11]. In another study that examined rates of chronic health conditions by race and ethnicity in children, Hispanic children were more likely to report visual impairment compared to white and black children [16].

Based on estimates from National Survey of Children’s Health (NSCH) 2011–2012, The National Center for Children’s Vision and Eye Health Report 2016 states that nearly 83% of children between ages of 6 and 11 years had their vision tested within two years of the survey. Screening rates varied by household income, insurance coverage, ethnicity and educational status of adults in the household. Children from low-income household were less likely to receive vision screening (62% versus 72%). Screening rates of children where family had private insurance was 72% compared to 58% where families were uninsured. Only 52% of children from households with parents with less than high school level of education were screened, in comparison to 72% children from households where adults had a greater than high school level of education. Only 57% of children identifying themselves as Hispanic received vision screening as compared to 72% white non-Hispanic and 71% black non-Hispanic children. Data on follow-up rates and those receiving treatment was either not easily available or was unreliable due to lack of standardized procedures [11]. School-based vision and hearing screening can be an effective way to detect early visual and auditory impairments and remove barriers to academic success at school only if adequate follow-up is available [17].

### 1.2. Background: Effectiveness of School-Based Screening Programs/Evidence from the Literature

We conducted a comprehensive literature review of currently published peer reviewed journal articles, policy statements, and publications by national institutions, using various informational sources such as Google Scholar, PubMed, and the Socrates database provided by Stanford University library services to comprehend the importance of school-based screening programs.

#### 1.2.1. The Role of School Nurse in Screening Programs

According to the National Association of School Nurses, (NASN), school nurses are required to conduct screenings, referrals, and follow-up activities in schools as part of their role to provide secondary prevention services for health concerns that can be detected early and treated in a timely manner [18]. A systemic review of role and impact of school nurses reported that vision screening and referrals provided as part of health promotion and disease prevention efforts, were some of the most promising services provided by school nurses in American elementary schools and approximately 66% of referrals to school nurses were for screening [19]. 

A study from eight New York schools with school-based vision programs reported increased usage of corrective glasses in classrooms after students screened for vision problems were followed up and encouraged to wear glasses at school by their teachers [20]. In 139 Indiana schools, students from lower median family incomes screened for vision problems were more likely to get referrals to eye care providers (referral rate 14.6%) compared to students with higher median family income (referral rate 8.5%). Students that failed screening tests in lower grades were more likely to perform poorly in standardized academic tests in higher grades. Additionally, increased demand on school nurses, lack of school resources and diversity of socio-economic status made compliance with vision screening requirements and follow-up challenging [21]. In 10 North Carolina schools, about two-thirds of the students with abnormal vision screening received documented follow-up with an optometrist or ophthalmologist when school nurses were actively involved in regular follow-up with parents and providers [22]. 

#### 1.2.2. Other Models of Providing School-Based Vision Programs

A systemic review conducted by Knopf et al. concluded that School-Based Health Centers (SBHC) can be effective in improving educational and health outcomes in low income and disadvantaged communities by providing primary health care services to students and their families, including vision screening and follow-up [23]. An example of this model is the first self-sustaining school-based vision center operated by Cincinnati Health Department, Ohio, which provided comprehensive follow-up services including eye exams, fitting and adjusting eyeglasses, and medical eye care to thousands of school students on government-assisted insurance programs through a public-private partnership [24]. Evolving models for providing vision screening and follow-up care in community settings include mobile eye clinics [25], use of portable equipment, including optical shop that can be brought to the school premises, and enlisting the help of volunteers, parents, teachers, and ophthalmic technicians to assist in the screening process. A school-based program run by the county health department in Toledo, Ohio arranged for professionals like optometrists and local businesses that provide eyeglasses to come directly to the schools to offer vision care services [6]. An innovative school-based screening program called EyeSpy tested vision while children played adventure games on a computer. More recently parents are being encouraged to conduct an initial screening at home using validated charts from national- or international-level blindness prevention organizations. Other instruments currently under validation study include smart phone-based apps to test visual acuity and instrument- based screening. 

#### 1.2.3. Vision Screening Follow-Up and Referral

Although accurate rates of follow-up and treatment after a failed school vision test are not readily available, reported follow-up and treatment rates range between 5% and 18% [6,25,26]. Many students referred for follow-up, especially minority and urban youth and children from low socio-economic status, may not receive the care they need due to multiple reasons, including lack of understanding on parents’ part, parental unawareness of results, miscommunication and lack of clarity in communicating screening results, delay in administering follow-up care, financial problems, lack of insurance, social and family issues such as inability to take time off from work, logistical problems such as scheduling issues and perceptual barriers of parents, parental mistrust of the school nurses, and parental misunderstanding of follow-up procedures [6]. Other barriers include transportation cost difficulty, access to transportation, payment difficulty, lack of commitment, and lack of means of communication [26]. 

Diao et al. reported that in Philadelphia, when a mobile ophthalmic unit equipped like a pediatric ophthalmologist’s office visited parking lots of elementary schools in predominantly socioeconomically disadvantaged communities, 82 of the 132 children who failed an eye exam and were referred for follow-up with parental consent received an ophthalmic consultation. The improvement in follow-up rate from an earlier rate of 53% to 62% was statistically significant. The school nurses involved in the follow-up reported high satisfaction with the program due to its simpler logistics, convenience and effective use of their time [25]. Other studies reported successful follow-up and treatment of vision problems through community outreach programs in inner city areas [27] and social worker intervention [26].

Focus groups with students, parents, and teachers in a school-based program that provided free corrective lenses to low-income students in Los Angeles, CA through a mobile eye clinic confirmed that after receiving corrected lenses, student attention, class participation, and academic skills improved. Students reported better attention, fewer headaches, and feeling of wellbeing, and adults stated that provision of free services in schools removed financial and psychological barriers to students receiving corrective lenses [5].

Overall, the result of the different interventions could be described as having moderate success, with reports of less that 70% of follow-up. Here, we describe a screening intervention embedded in a quasi-experimental project that consisted in placing full-time nurses in four demonstration schools in San Jose, California, for a five year period, and provide a descriptive analysis of the follow-up rates in demonstration and comparison schools.

## 2. Materials and Methods

### 2.1. Screening and Follow-Up Intervention in San Jose Unified School District, California

As part of the San Jose Unified School District (SJUSD) School Nurse Demonstration Project funded by the Lucile Packard Children’s Hospital (LPCH) and the Lucile Packard Foundation for Children’s Health (LPFCH), full-time school nurses were placed in four SJUSD schools, serving children from kindergarten through to grade 8 over a five-year period (2007–2012), and a nurse practitioner was recruited at School Health Clinics of Santa Clara County. The age of the children ranged between 5 and 13 years of age. One of the goals of the project was to assure that school nurses were involved in screening for vision, referrals to health care providers, and follow-up with families to ensure that the students received the treatment they needed.

Four demonstration schools (two elementary and two middle schools serving around 2800 children each year) with full-time nurses were matched with five comparison schools (four elementary and one middle school, serving around 3445 children each year) where nurses were hired on a part-time basis, as was customary. All the nurses were equally trained to perform vision and hearing screenings and customary follow-up, but only full-time nurses in demonstration schools were able to provide comprehensive follow-ups and care coordination with other health care providers [28]. 

The schools were not randomly selected. Demonstration schools with full-time nurse were selected on ethical grounds to provide additional support to the most needy schools in the SJUSD. Demonstration schools included 83% low-income students as calculated by eligibility for federal free and reduced-price lunch program compared to 68% in the comparison schools—which were selected among the most similar to the demonstration schools in the district. As described in Table 1, 81% of students in demonstration schools identified themselves as Latino/Hispanic, compared to 70% in comparison schools. Academically, a higher number of students in comparison schools scored proficient in English language arts and math when compared to students in demonstration schools.

Nurses in demonstration schools were present 5 days per week and were hired as 1.0 full-time equivalent (FTE), with nurse-to-student ratio in elementary schools of about 1:550 and approximately 1:1000 in middle schools. Nurses hired at comparison schools worked an average of 1.7 days per week (0.34 FTE) at each school and had higher turnover rate. Nurses were involved in screening for vision, referrals to health care providers and follow-up with families to ensure that the students received their glasses.

Here, we focus first on illustrating the impact that having full-time nurses in schools—with large numbers of economically vulnerable children—had in increasing follow-up to vision screening. Then, we assess teachers’ perceptions on the impact of having school nurses providing follow-up and appropriate treatment. Other results of the overall nurse demonstration project have been reported elsewhere [28,29].

### 2.2. Summary of Evaluation Design and Population Sample

Within the mixed methods, a quasi-experimental evaluation used to compare the four schools with full-time nurses with the five with part-time nurses in SJUSD. In this study of vision screening, we first collected descriptive information on screening rates and follow-up. The study population included all students who received vision screening in the schools between 2007 and 2012. Secondly, we analyzed relevant questions from a 2013 online survey of all the teachers in the participant schools.

### 2.3. Data Collection Instruments and Analysis

Nurse monitoring and tracking tools were used to keep records of the number of students screened for vision, referred for professional examination and evaluated by a vision specialist. Parents of all students that failed the vision test in both demonstration and comparison schools were sent a referral letter and contacted by phone by the school nurse and health office support staff to follow up with the eye doctor for their child’s eye examination. The school nurses in the demonstration group followed up with additional contact efforts and reported that it took an average of three contacts with the parents before students were seen by an eye doctor. In several cases, it took seven or more follow-up attempts before the parents complied. We did not have access to personal identifiers to link the tracking data to personal characteristics, and we were only able to perform a descriptive analysis of screening rates and follow-up. We discuss this limitation in our discussion and conclusion sections.

We also conducted an online teacher survey, and all 160 teachers in demonstration schools and 171 teachers in comparison schools were prompted to respond. The purpose of the survey was to elicit teacher responses about their perceptions of the work being done by the school nurses and their perceived impact of having a full-time nurse at their school. The questionnaire consisted of 25 items, including 16 multiple choice questions, six open-ended questions, and three questions with both multiple-choice and free response options. A comprehensive quantitative and qualitative analysis of the survey questions was published in an earlier paper [29]. Here, we include only responses relevant to vision screening. The specific open-ended questions in which teachers commented about their perceptions of the screening intervention that we analyze here are: (1) “If you referred students to the school nurse during the year, what was the reason for your referral?” and (2) “In your opinion, what is the most beneficial activity performed by the nurse at your school?” To analyze the data we used an open and axial coding method to develop themes and categories through an iterative process based on teacher responses to open-ended questions [30]. The data was independently analyzed by two researchers. In the event of a difference of opinion regarding the theme of an open-ended response, a third reviewer resolved the conflict, and the response was recorded accordingly. Details on the qualitative analysis have been reported elsewhere [29].

## 3. Results

**Figure 1:**
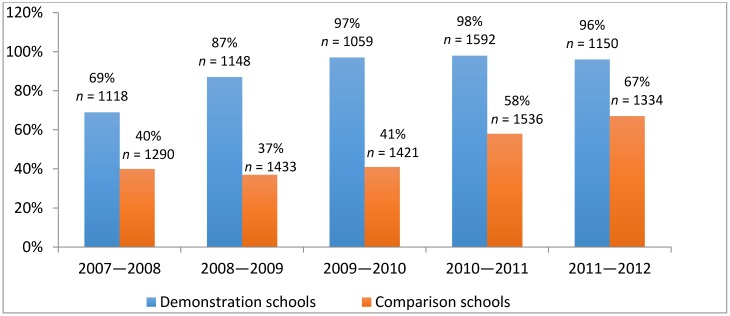
Percentage of students with vision problems examined by vision specialist after nurse referral (*n* = Actual number of students who were screened for vision that year).

Overall, our results provide evidence that, when full-time nurses are available at schools, follow-up rates are much higher than those reported by other types of programs and described in our literature review. First, we analyzed the rates of screening and follow-up based on monitoring data collected by school nurses between 2007 and 2012. As described in Figure 1, after full implementation of the project, the follow-up rates in demonstration schools ranged between 96% and 98% in comparison to the pre-intervention year of 2007–2008. The follow-up rates in comparison schools remained between 41% and 67%.

In the demonstration schools, 1150 students underwent vision screening (425 in elementary and 725 in middle schools) in the 2011–2012 academic year, and a similar number of students were screened in any of the previous years, as noted in Figure 1. In 2011–2012, a vision specialist evaluated 224 of the 234 students referred by nurses in demonstration schools for possible vision problems, indicating a nurse referral rate of 20%, and a follow-up rate of 96%. In comparison schools, 1334 students (913 in elementary and 421 in middle schools) were screened, and a vision specialist examined 95 of the 142 students referred for vision problems, indicating a nurse referral rate of 10% and a follow-up rate of 67%. Of those screened, students in kindergarten, grade 3 and grade 6 had mandatory screening, and students in other grades were screened based on teacher referral. Within demonstration schools, rates of student follow-up by vision specialist were similar for elementary and middle schools, but follow-up rates declined in middle schools in comparison schools. In total, we analyzed data of 6067 students who were screened in demonstration schools with full-time nurses between 2007 and 2012, and 7014 in comparison schools with part-time nurses during the same period. 

Second, we analyzed qualitative data collected in 2013 through online teacher surveys for two specific questions. 59 teachers in four demonstration schools and 70 teachers in five comparison schools participated in the online survey, which reflects a response rate of 37% in demonstration schools and 41% in comparison schools. 

### 3.1. Question 1: “If You Referred Students to the School Nurse during the Year, What Was the Reason for Your Referral?”

In the qualitative analysis, 37 teachers in demonstration schools and 40 teachers in comparison schools responded to this question. While teachers referred students to the school nurses for a variety of reasons, 38% of teachers in the demonstration schools indicated that they referred their students for vision screening, follow-up, and support. Yet, only 10% of teachers in comparison schools with part-time nurses reported referring students for vision screening and follow-up.

### 3.2. Question 2: “In Your Opinion What Is the Most Beneficial Activity Performed by the Nurse at Your School?”

A total of 53 teachers in demonstration schools and 56 teachers in comparison schools responded to this question. In the demonstration schools, 24.5% of teachers reported that vision and hearing screenings and follow-up were of the most beneficial activities performed by school nurses. In the comparison schools, only 10.7% teachers reported vision screening and follow-up among the most important activities performed by the nurses at their schools. Table 2 illustrates some of the feedback by teachers describing the benefits of screening provided by nurses. Overall, teachers in demonstration schools were very satisfied with the presence of full-time nurses in their schools, and teachers in comparison schools expressed the need for more nurse time at their schools.

In summary, our results indicate that the efficacy of follow-up for vision screening improved in schools with full-time nurses who had more time to focus on follow-up. Not only was the intervention highly successful, but it also achieved greater level of follow-up than reported in most previous studies done in school settings.

## 4. Discussion

Conducting timely screening tests for vision and hearing in school-based settings can especially benefit low-income students who may otherwise lack health insurance and access to care [31]. This study shows that having fulltime nurses in schools provides higher rates of follow-up than other methods previously used [20,21,22]. Having school nurses ensures appropriate referrals and follow-up with health professionals and can also avoid occurrence of chronic health problems and disability for these children [22].

In general, quasi-experimental studies are exposed to potential bias, but they are the methodology of choice when programs are funded and implemented to serve the most vulnerable groups and ethical considerations preclude randomization. In this particular study, the demonstration schools had a slightly higher number of low income and minority students than the comparison schools. Over the course of the project, any materials developed to support students in the demonstration schools were also shared with all the nurses in the SJUSD. This might have introduced a conservative bias in the sense that some of the benefits of having full-time nurses in some schools could also be felt in the rest of the district and could partly explain why we also observe a modest improvement in follow-up practices in the comparison schools. We also must caution about generalizing our findings, as we do not use inferential statistical analysis and our sample is limited to schools in California.

There is limited literature available on the cost-effectiveness of school-based vision screening programs and students’ acceptability of wearing prescription glasses at school. Yet, there is a growing need to offer the services so that all students get an opportunity to achieve at school [32]. Since low-income children have a disproportionately higher incidence of vision problems that may affect their academic performance, schools should encourage health policies that address common barriers to children receiving glasses and wearing them at home and at school [33].

School authorities should not only target improving screening rates to reach recommended national levels and ensure standardized quality health screenings, but also to follow up with parents and health care professionals. Trained individuals performing screenings following a rigorous protocol should also develop specific procedures to inform families about both normal and abnormal results, and encourage appropriate follow-up of referrals with health care providers [34]. As demonstrated in this study, school nurses can coordinate care and work with students and families to connect them to appropriate health care professionals and community services, even to a greater extent than has been previously reported [35].

## 5. Conclusions

Having full-time nurses at schools is a successful way of assuring that children identified as needing care actually receive it. Schools provide an ideal setting to conduct health screenings, and school nurses can play a pivotal role in providing these preventive services in a timely manner [36]. The objective of the study was to describe nurse referral and follow-up rates, yet it would be useful to know the diagnosis and treatment once the students were seen by a physician or eye specialist, and to investigate if all students were equally likely to receive treatment once a diagnosis was made. This would require accessing clinical data, which was not available in this study. Overcoming such limitations in future work could open the door to rigorous cost-effectiveness analysis. In the meantime, this work provides evidence that school-based health screening services for low-income students can have appropriate follow-up if nurses are properly trained and available in the school.

## Figures and Tables

**Table 1:** Student demographics in demonstration and comparison schools in 2011–2012.

School Characteristics	Demonstration Schools in 2011–2012	Comparison Schools in 2011–2012
Total students enrolled	2785	3445
Students enrolled in free or reduced-price lunch	83.3%	68.2%
Latino/Hispanic Students	81.1%	70.2%
English language learners	44%	47%
Proficient or advanced in English language arts	42%	46%
Proficient or advanced in mathematics	41.2%	53.6%

Source: Education data retrieved from http://www.ed-data.k12.ca.us/Pages/Home.aspx. Accessed on 13 July 2017; data provided by the San Jose Unified School District (SJUSD) data warehouse [28].

**Table 2:** Examples of quotes of teachers in demonstration school with full-time nurses about benefits of screening provided by nurses.

Yes! It is nice knowing that there is a nurse on campus that will follow through with the concerns and needs of the students. She is always willing to check up on students’ vision concerns and with medications.
To have eyes checked to see if the issues with reading were vision-related. To have her follow up on a health issue that has come up and I need some follow through from the parents. To have her contact the parents about medication for a child. To rule out any health issue that might be getting in the way of the child learning.
Our nurse has been very proactive in getting eye exams and glasses for students who need them. That certainly impacts their academic performance and makes my teaching more effective.
The nurse is a great resource for students with health problems. Also, she follows up when students have hearing and vision issues.
Everything! But the constant monitoring of vision and hearing testing has made a big difference for several of my students who have ended up getting glasses. I completely appreciate our nurse’s visibility and rapport with all our students. She is an important part of our school community!
At our school site, a large majority of students come from low-socioeconomic status homes which lack access to health services of any sort. The services provided at school are often the only access to support these students ever experience, and it’s priceless.
